# Ethical Challenges and Considerations in Dysphagia Management: A Scoping Review

**DOI:** 10.1111/1460-6984.70214

**Published:** 2026-02-27

**Authors:** Alida Naudé, Amisha Kanji

**Affiliations:** ^1^ Department of Health and Rehabilitation Sciences, Division of Speech‐Language and Hearing Therapy Stellenbosch University Cape Town South Africa; ^2^ Department of Speech Pathology & Audiology University of the Witwatersrand Johannesburg South Africa

**Keywords:** decision‐making, dysphagia, equity, ethics, informed consent, interdisciplinary care, patient autonomy, speech‐language therapy, telepractice

## Abstract

**Background:**

Speech and language therapists (SLTs) working in dysphagia care regularly navigate complex ethical dilemmas involving clinical risk, patient autonomy and cultural considerations. While ethical principles are well‐recognized in the field, consolidated evidence mapping how these principles and ethical reasoning components have been represented in the literature over time remains limited. This review offers a comprehensive synthesis of ethical challenges in dysphagia management across decades and contexts, uniquely structured using Rest's Four‐Component Model of ethical behaviour.

**Method:**

This scoping review was reported in accordance with the Preferred Reporting Items for Systematic Reviews and Meta‐Analyses Extension for Scoping Reviews checklist. A systematic search of databases from 1990 to 2024 identified 22 peer‐reviewed articles. Data were analysed using descriptive statistics and thematic content analysis, with ethical principles and components classified according to Beauchamp and Childress and Rest's Four‐Component Model.

**Main Contributions:**

Analysis revealed evolving ethical priorities within interdisciplinary roles and responsibilities of SLTs. These ethical priorities suggest the importance of ethical decision‐ making and person‐centred care in dysphagia management, specifically foregrounded by the focus on the principles of autonomy and informed consent and the components of moral judgement and sensitivity.

**Conclusion:**

The findings underscore the dynamic and complex ethical landscape of dysphagia management, emphasizing the need for cultural awareness and respect, shared decision‐making and evidence‐based practice. To navigate these challenges, SLTs require continuous education, interdisciplinary collaboration and adaptable ethical frameworks. This review provides a comprehensive synthesis of ethical challenges and considerations through longitudinal, theory‐informed analyses using Rest's Four Component model. This model is useful in breaking down complexity into understandable psychological steps, identifying gaps, guiding education and culture by fostering genuine ethical conduct beyond intellectual understanding across decades, geographic regions and professional roles in SLT.

**WHAT THIS PAPER ADDS:**

*What is already known on this subject*

- The field of dysphagia often places SLTs at the intersection of clinical care, patient preferences and ethical decision‐making, making the consideration of ethical principles an integral part of their practice.

*What this paper adds to existing knowledge*

- A comprehensive synthesis of ethical challenges and considerations in dysphagia spanning multiple decades, geographic regions and methodological approaches using Rest's Four‐Component Model to classify ethical components.

*What are the potential or actual clinical implications of this work?*

- Cultural awareness and shared decision making are vital aspects in dysphagia management, with the use of telepractice raising ethical concerns related to equity, privacy and informed consent.

## 1 Introduction

Dysphagia is a complex condition that requires speech and language therapists (SLTs) to balance multiple responsibilities, including assessment, diagnosis, intervention and management. Evidence‐based practice integrates patient perspectives, clinical expertise and research evidence, providing a structured framework for clinical decision‐making (Sackett et al. 1996). However, the emphasis on clinical safety from a clinician's perspective, often influenced by perceived legal liability and institutional risk aversion, can sometimes overshadow cultural, emotional and psychosocial considerations. This may result in care that is technically sound but ethically misaligned with person‐centred values. Recent literature has questioned long‐standing assumptions about aspiration risk. Common misconceptions in dysphagia management include the belief that prandial aspiration always warrants immediate restrictive intervention, that coughing during meals invariably signals physiological dysfunction and that thickened liquids consistently reduce aspiration without adverse effects.

These misconceptions reinforce the need to balance clinical recommendations with patient autonomy and quality of life (Palmer and Leslie 2025; Steele et al. 2021). This balance underscores the importance of ethical awareness and cultural, calling on SLTs to adopt more holistic, person‐centred approaches to dysphagia management, particularly when ethical challenges arise (Barnard et al. 2022).

Dysphagia management poses ethical challenges that extend beyond clinical protocols to encompass patient preferences, cultural identity, psychosocial well‐being and broader societal values. The act of eating and drinking is deeply embedded in social, cultural and familial traditions and decisions around texture modification, feeding alternatives or dietary restrictions can profoundly affect an individual's autonomy and quality of life (Barnard et al. 2022). In such contexts, clinical decisions are not solely about preventing aspiration or prolonging life, but also about supporting the person's sense of dignity, identity and inclusion, highlighting the need to ensure person‐centred care.

Several studies have explored ethical dimensions in speech‐language therapy (SLT), often framed within the broader concept of person‐centred care (Epstein and Street 2011; Naudé et al. 2025). This approach prioritizes shared decision‐making, respect for patient values and collaborative goal setting. In dysphagia management, person‐centred care requires clinicians to consider the balance between clinical safety and personal choice, especially in complex cases such as end‐of‐life care or when cultural food preferences conflict with clinical recommendations (Palmer and Leslie 2025).

Ethical principles, as outlined by Beauchamp and Childress (2019)—autonomy, beneficence, non‐maleficence and justice remain foundational in guiding SLT practice when complex cases arise. These principles provide a framework for navigating difficult decisions. Difficult decisions may include when to override a patient's wishes for safety concerns, how to ensure equitable access to dysphagia services across linguistic and socioeconomic barriers and when to prioritize comfort over prolonging life. However, the application of these principles is often nuanced and requires clinical reasoning that accounts for both patient and systemic factors (Leslie and Lisiecka 2020).

Autonomy and beneficence are principles that frequently intersect, requiring clinicians to weigh the risks of aspiration against a patient's right to enjoy food in a meaningful way (Sharp and Wagner 2007). Case‐based studies have been particularly useful in illustrating how ethical principles are applied in practice. For example, Gunasekaran et al. (2024) demonstrated how clinical reasoning in acute care dysphagia management is influenced by institutional protocols, time constraints and interprofessional communication. Tohara et al. (2003) and Vose et al. (2018) explored decision‐making frameworks in acute medical settings, emphasizing the importance of interdisciplinary collaboration. Pillay and Pillay (2021) highlighted the complexities encountered in under‐resourced settings in South Africa, where limited access to diagnostics and rehabilitation services often forces SLTs to make ethically challenging decisions with constrained information.

Despite these contributions through case‐based studies, a gap remains in the systematic exploration of how SLTs engage with ethical components—the underlying psychological processes that influence ethical behaviour and how SLTs make the ethical decisions related to the ethical principles outlined by Beauchamp and Childress (2019). To address this gap, this scoping review employed Rest's Four‐Component Model (1994), a widely accepted framework in bioethics and professional ethics education. Rest identified four interrelated processes required for ethical action:

1. **Moral (or ethical) sensitivity**—The ability to recognise the presence of an ethical dilemma and understand how one's actions affect others. In dysphagia care, this may involve recognising how a standard recommendation could unintentionally violate a patient's cultural or personal values.
2. **Moral (ethical) judgment**—The ability to decide the most ethical course of action among competing options. This is particularly relevant when weighing aspiration risk against the enjoyment of food, or when institutional protocols conflict with a patient's expressed wishes.
3. **Moral (ethical) motivation**—The prioritisation of ethical values over other competing interests (e.g., workload, institutional norms, fear of litigation). For example, advocating for a patient's preferred feeding plan despite concerns from other professionals.
4. **Moral (ethical) courage (or action)**—The ability to act ethically despite barriers, such as professional disagreement or organisational pressure. This is critical when SLTs must advocate for patients whose choices might be seen as ‘non‐compliant’ by medical staff.

Despite its wide uptake in healthcare ethics education and professional ethics research, Rest's Four‐Component Model has been underutilised in SLT ethics, including dysphagia‐related scholarship. Existing SLT literature has largely focused on identifying ethical principles or describing ethical dilemmas, often through philosophical discussion or case‐based reflection, without systematically examining the psychological processes that underpin ethical behaviour in practice. This principle‐centred emphasis means that ethical reasoning is frequently conceptualised in terms of what clinicians ought to do, rather than how ethical awareness, prioritisation and action are developed and enacted in real‐world clinical contexts. As a result, components such as moral motivation and moral action—critical for advocacy, risk‐aligned decision‐making and ethical courage—remain comparatively underexplored in SLT ethics literature. Applying Rest's Four‐Component Model therefore addresses a substantive gap by offering a process‐oriented framework that compliments principle‐based ethics and enables a more comprehensive understanding of ethical practice in dysphagia management.

Rest's model complements the principled approach of Beauchamp and Childress by shifting the focus from what is ethical to how ethical decisions are recognised, reasoned and enacted in practice. Its use in this scoping review enables a more process‐oriented understanding of SLT behaviour in ethically complex dysphagia scenarios.

Furthermore, in reviewing the literature, it is evident that the ethical challenges faced by SLTs are not uniform across settings. In low‐ and middle‐income countries (LMICs), ethical decision‐making is often compounded by systemic inequities, limited access to specialist services and cultural norms that shape patient expectations and caregiver roles (Pillay and Pillay 2021). Yet, few studies from LMICs have been included in past ethics reviews, resulting in a predominantly Western framing of ethical practice. By incorporating international studies from countries such as South Africa, India and Brazil, this scoping review aims to broaden the discourse and acknowledge the global relevance of ethical reflection in dysphagia care.

Finally, while some prior reviews (e.g., Sharp and Wagner 2007; Arvedson and Lefton‐Greif 2007) have discussed ethical issues in paediatric or palliative contexts globally, they often lack a unifying theoretical framework and focus on isolated principles or cases. This scoping review offers a comprehensive synthesis by spanning multiple decades, methodological paradigms (philosophical and social scientific) and global contexts. Through the application of Rest's model, it moves beyond identifying principles to examine how ethical practice is enacted in real‐world SLT settings.

## 2 Methodology

This study was conducted as a scoping review with an embedded deductive qualitative analytic component. While the primary purpose of the scoping review was to systematically map the breadth, characteristics and trends of the literature on ethics in dysphagia management, Rest's Four Component Model of Moral Behaviour (Rest 1986) was applied deductively as a theoretical lens during data extraction and analysis. This approach enabled structured classification of ethical components across studies while remaining consistent with the exploratory and mapping aims of a scoping review. Rest's model proposes that ethical behaviour involves four psychological processes: moral sensitivity (recognizing the presence of an ethical issue), moral judgment (determining the right course of action), moral motivation (prioritizing ethical values over competing interests) and moral character or action (following through on ethical intentions despite challenges). This model offers a nuanced understanding of how ethical reasoning unfolds in real‐world practice.

Rest's framework aligns conceptually with the principle‐based approach of Beauchamp and Childress (2019), which outlines the four core ethical principles as foundational in clinical ethics. While the principles provide the content of ethical reasoning, Rest's model provides the process through which such reasoning is enacted. Together, these frameworks offer a comprehensive lens through which to examine the ethical reasoning and behaviour of speech‐language therapists (SLTs) in dysphagia care.

### 2.1 Search Strategy and Selection Criteria

This scoping review was reported in accordance with the Preferred Reporting Items for Systematic Reviews and Meta‐Analyses Extension for Scoping Reviews (PRISMA‐ScR) checklist by Tricco et al. (2018).

In December 2024, we conducted a comprehensive literature search to identify peer‐reviewed publications addressing ethics within the SLT discipline for the period spanning 1980 to 2024. The decision to commence the search from 1980 was driven by the transition to electronic record‐keeping in library databases. Before 1980, articles were exclusively available in hard copy, and our search focused on the electronic database era to ensure a thorough and efficient retrieval of relevant literature.

Our search strategy incorporated a comprehensive range of terms related to SLT and ethics. Due to the interchangeable use of the terms ‘ethics’ and ‘morality’ in the literature, and the inconsistent application of ‘ethics’ as a keyword, we included a broad set of ethical concepts and values. Truncation and Boolean logic were employed to maximise search sensitivity across five databases: PubMed, Scopus, CINAHL, PsycINFO and Web of Science. An example of the Boolean search string (as applied in PubMed) is as follows: (‘speech‐language therapy’ OR ‘speech‐language pathology’ OR ‘speech therapy’ OR ‘communication disorders’ OR SLP OR SLT) AND (ethic* OR moral* OR ‘informed consent’ OR autonom* OR ‘moral reason*’ OR ‘moral judgment’ OR justice OR paternalism OR confidentiality OR care OR duty OR responsibility OR discrimination OR attitud* OR value* OR ‘best practice’ OR ‘problem solving’ OR ‘decision making’)This syntax was adapted as needed to meet the indexing and search functionality of each specific database (e.g., subject headings in CINAHL and MeSH terms in PubMed). The aim was to identify peer‐reviewed articles that engaged with ethical concepts in the context of dysphagia or SLT more broadly, allowing for diversity in terminology, theoretical orientation and methodology.

All screening, coding and categorisation were conducted independently by both authors. Any discrepancies—whether related to article inclusion, assignment of ethical principles or classification using Rest's Four‐Component Model—were resolved through collaborative discussion. In cases of initial disagreement, the reviewers revisited the relevant literature and coding criteria until consensus was reached. This consensus‐building process contributed to the trustworthiness and consistency of the review findings.

**FIGURE 1:**
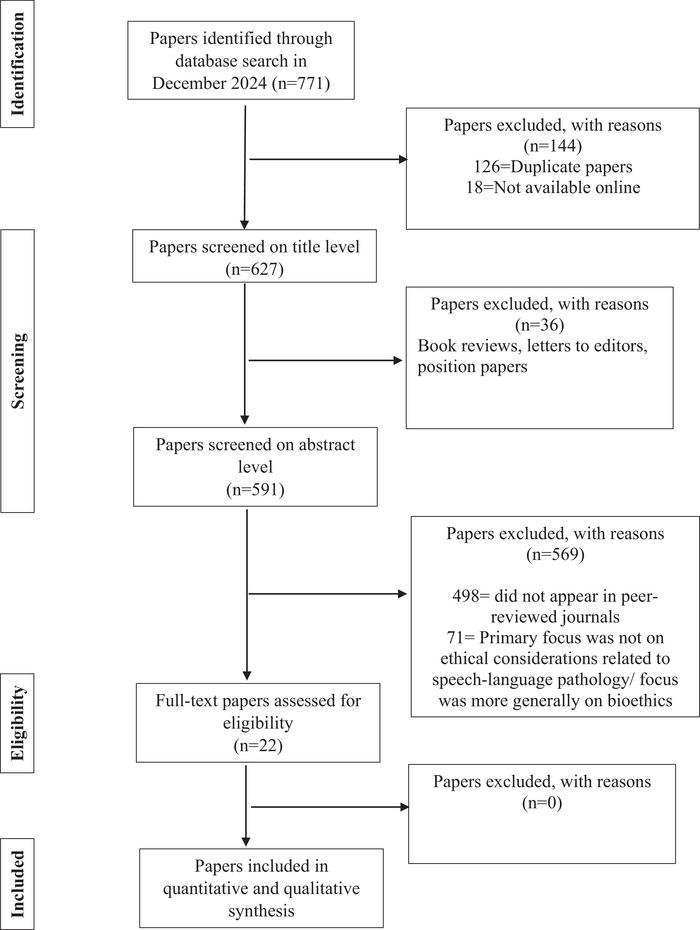
The Preferred Reporting Items for Systematic Reviews (PRISMA) and Meta‐Analyses flowchart with search results and reasons for exclusion.

The search yielded a total of 771 papers. After removing 126 duplicates, a total of 645 records remained. Eighteen papers were excluded at this stage because they were only accessible in hard copy and not available online, resulting in 627 records screened at the title level. During title screening, 36 records were excluded. The remaining 591 titles were assessed for relevance, and 93 papers were identified as being published in peer‐reviewed journals, a requirement for inclusion. Abstract screening of these 93 papers resulted in the exclusion of 71 papers. Only English‐language articles were included in this review. Non‐English records were excluded due to the lack of reliable translation resources available to the research team. Inclusion and exclusion criteria are further outlined in Table 1. A PRISMA flow diagram (Figure 1) summarizes identification, screening, eligibility and inclusion decisions.

**TABLE 1:** Inclusion and exclusion criteria for study selection.

Inclusion criteria	Exclusion criteria
Peer‐reviewed empirical studies	Non‐peer‐reviewed literature (e.g., opinion pieces, editorials, book reviews)
Focus on ethical principles, challenges or considerations in dysphagia management	Studies not primarily focused on ethics in speech‐language pathology
Studies involving speech‐language pathologists or related professionals	Studies focused exclusively on bioethics or general healthcare ethics
Published between 1980 and 2024	Studies only available in hard copy (not electronically accessible)
Articles written in English	Non‐English articles

### 2.2 Data Extraction and Analysis

The authors used a structured data extraction form to collect variables from the selected studies. The structured form included operational definitions for ethical principles, topics, Rest components and SLT roles which guided the extraction of data. The authors jointly conducted a pilot extraction to ensure consistency. Following this, each author extracted data from all 22 included studies and discussed any discrepancies which were resolved through consensus. As this was a scoping review, no formal quality appraisal of individual studies was performed. We employed a two‐phase research methodology that combined quantitative and qualitative techniques to extract and analyse information from the 22 papers.

In the quantitative phase, descriptive methods were employed to analyse the included publications based on the following categorical variables:

1. Publication metadata, including author(s), journal and country of origin. For the purpose of geographical analysis, the country of origin for each study was determined based on the institutional affiliation of the first author at the time of publication. This approach was selected to ensure consistency and replicability, as the first author is typically responsible for the primary conceptual and analytical contributions. In instances where the first author's affiliation spanned more than one country, the primary listed affiliation was used. Studies were classified as ‘global’ if they featured author affiliations from multiple countries without a clear primary national context or if the content addressed cross‐cultural or multinational themes that were not tied to a specific country. This category was used to capture the increasing trend of international collaboration and globally relevant discourse in ethical dysphagia care. As part of the analytic process, authorship patterns were reviewed to identify potential overlap across studies. Three author groups appeared more than once within the dataset, reflecting longitudinal scholarly engagement and possible duplication of conceptual frameworks or participant populations.
2. Temporal distribution, by decade (1980s, 1990s, 2000s, 2010s and 2020s).
3. Research approach classified as either philosophical or social scientific. Papers were coded as philosophical when their primary purpose was conceptual, normative or principle‐driven, focusing on ethical theory, ethical principles or conceptual analysis without presenting a specific clinical case as the central analytic unit. Papers were coded as case‐based when ethical issues were examined through one or more concrete clinical scenarios, vignettes or practice‐based examples, with ethical reasoning embedded within applied decision‐making contexts. These categories were defined a priori and applied deductively during data extraction based on the dominant methodological orientation of each paper. Where ambiguity arose, categorisation was resolved through reviewer consensus.
4. Ethical focus, categorised according to:
  - Ethical topics (e.g., informed consent, confidentiality, conflict of interest, research integrity, record keeping, risk management and veracity), based on Berkovic (2023). Topic focus was coded using a primary‐topic assignment approach. Although many studies addressed multiple, conceptually overlapping ethical concepts (e.g., autonomy and informed consent), each study was assigned a single dominant topic based on its central analytic emphasis, as reflected in the study aim, research questions and primary discussion. This approach was adopted to support clarity in descriptive reporting and to enable meaningful trend analysis without double counting studies across multiple topic categories. In one instance, a study was coded as having a dual topic focus where two ethical issues were equally central and analytically inseparable. All topic‐coding decisions were applied deductively and resolved through reviewer consensus.
  - Ethical principles as outlined by Beauchamp and Childress (2019).
  - The professional role of the speech‐language therapist (SLT) was coded deductively using established role categories described by Stach (2010), including assessment, management, counselling, advocacy, education, research, consultation and administration. These categories were selected a priori as they reflect internationally recognised domains of SLT practice and are commonly used to describe professional scope across clinical and service‐delivery contexts. Each study could be coded for more than one SLT role where applicable, based on the activities and responsibilities described in the paper. Coding decisions were resolved through reviewer consensus. Ethical decision‐making components, based on Rest's Four‐Component Model (1994). Coding of ethical components using Rest's Four‐Component Model did not require explicit reference to the model or its terminology within the original studies. Instead, components were identified based on conceptual and functional alignment with Rest's definitions. For example, moral sensitivity was coded when authors described recognition of ethical dilemmas or awareness of ethical implications; moral judgment when ethical reasoning, weighing of options or decision‐making processes were articulated; moral motivation when prioritisation of ethical values over competing pressures was evident; and moral action when authors described the enactment of ethical decisions despite contextual or institutional barriers. Given that these constructs are often implicit in applied ethics literature, coding decisions were based on the dominant analytic emphasis of each paper and were resolved through reviewer consensus.).The term *ethical* is used interchangeably with *moral* in this study (Clarkeburn 2002).

Each of these categorisations is explored in detail in the results and discussion sections to provide a comprehensive understanding of ethical discourse in dysphagia management.

### 2.3 Temporal Analysis

Temporal analysis was conducted by grouping included studies by decade of publication (1990s, 2000s, 2010s and 2020s) and comparing the frequency and distribution of ethical principles, ethical components (Rest's model), and SLT roles across these periods. This descriptive, comparative approach was used to identify patterns of continuity and change over time, consistent with the exploratory aims of a scoping review.

### 2.4 Trustworthiness

To enhance the rigour and reliability of the findings, we applied the trustworthiness criteria outlined by Brantlinger et al. (2005), using Braun and Clarke's (2022) framework to guide the thematic analysis process. We provide the following examples of how creditability, confirmability, dependability and transferability were addressed.

Credibility was established through multiple rounds of discussion between the two authors, where interpretations were critically examined and codes refined until consensus was reached. For example, during the classification of Rest's Four‐Component Model, disagreements about whether a study reflected ‘moral sensitivity’ versus ‘moral motivation’ were discussed and resolved by revisiting the definitions with illustrative quotes from the articles. Reviewer positionality contributed to credibility: both are SLTs registered with the Health Professions Council of South Africa and bring complementary expertise—one with 23 years of clinical practice and ethics training, and the other with 14 years of experience in both clinical and academic settings.

Confirmability was supported by maintaining an audit trail that included documented coding decisions in a shared spreadsheet, memos written during theme development, and version‐controlled files with tracked changes. For example, coding matrices were updated iteratively and retained for verification by an external reviewer not involved in the initial analysis.

Dependability was ensured by following a structured protocol for both the quantitative and qualitative phases. Decisions were logged systematically—for instance, we recorded why certain ethical principles were assigned to specific papers and how we interpreted evolving ethical themes across decades. Our analysis process logs and decision trees enable another researcher to retrace our steps.

Finally, transferability was enhanced by providing rich descriptions of the study context, including the professional background of the reviewers, and the global scope of the included literature. For example, we note how ethical challenges differed between high‐income and LMICs, supporting readers in determining applicability to their own settings. and methodologies, enabling other researchers to evaluate the applicability of the findings in different settings. This comprehensive approach ensured that the findings of this study are robust, reliable and reflective of the ethical considerations pertinent to dysphagia management in SLT.

## 3 Results

### 3.1 Overview of Included Studies

The scoping review identified 22 articles meeting the inclusion criteria, published between 1990 and 2024. Six of these were from the 1990s, seven from the 2000s, three from the 2010s and six from the 2020s. The number of studies fluctuated between the 2000s and 2020as. However, due to the small sample of studies, these temporal changes should be interpreted with caution.

The journals represented in the review underscore the interdisciplinary nature of the research on ethics in dysphagia management. Most contributions came from SLT‐specific journals (11 studies); followed by rehabilitation journals (six studies). Two studies were from bioethics related journals and one study from an interdisciplinary journal.

Geographically, the research was mostly conducted in high‐income countries. The United States followed by Australia. In one article, the first author held institutional affiliations in both the United States and Australia; however, for consistency and replicability, the study was classified according to the primary institutional affiliation of the first author. This accounts for the apparent discrepancy between the number of articles and the number of countries represented. Smaller contributions came from the UK/Ireland (and Brazil. Additionally, two studies were categorized as global, as they involved international collaborations with co‐authors from multiple countries and did not indicate a single primary geographic context for the research.

### 3.2 Authorship Patterns and Overlap

Authorship patterns showed overlap, with three recurring author groups contributing multiple studies. Sharp and colleagues (1996–2007) produced a series of principle‐based analyses on autonomy, beneficence and consent in dysphagia care, shaping early ethical discourse. Lefton‐Greif and Arvedson (1997–2007) focused on paediatric feeding, integrating family‐centred and justice‐oriented frameworks. Hemsley and Balandin (2003) addressed communication access and equity, linking ethics to contemporary issues like telepractice. While these groups add thematic depth and continuity, repeated theoretical framing may limit diversity of perspectives. Future reviews should account for this overlap when interpreting recurring arguments.

### 3.3 Ethical Approaches

The reviewed studies employed two primary ethical approaches: philosophical and case based. Of the 22 articles, 59% (*n* = 13) adopted a philosophical approach, focusing on ethical principles such as autonomy, beneficence, non‐maleficence and justice. These studies provided theoretical frameworks that guided clinicians in understanding and navigating ethical complexities. In contrast, 41% (*n* = 9) of the studies used a case‐based approach, offering practical insights derived from real‐world scenarios to address specific ethical dilemmas encountered in clinical practice.

Across decades, both philosophical and case‐based approaches were evident in the literature. In the 1990s, philosophical analyses slightly outnumbered case‐based studies, with 4 out of 6 studies adopting a principle‐based or conceptual approach and 2 presenting case‐based analyses. However, from 2000 to 2010, there was an increase in case‐based papers (*n* = 5), compared to 2 philosophical papers during the same period. A similar pattern continued in the 2010s (2 case‐based, 1 philosophical) and in the 2020s (4 case‐based, 2 philosophical).

### 3.4 Topic Focus

The studies included in this review addressed a wide range of topics, reflecting the complexity of ethical considerations in dysphagia management. All but one study was assigned a single dominant topic focus, in line with the primary topic coding approach described in the Methods. Autonomy in dysphagia care was the most frequently addressed theme, appearing in 32% (*n* = 7) of the studies, followed by informed consent, which was explored in 27% (*n* = 6) of studies.

Ethical dilemmas in dysphagia care were discussed in 27% (*n* = 6) of the studies, focusing on the challenges SLTs face in balancing clinical safety with patient preferences. Similarly, dysphagia management after stroke, addressed in 18% (*n* = 4) of studies, explored the ethical challenges in balancing aspiration risk with quality of life—particularly in the context of cognitive or communication impairments that may affect informed decision‐making. Emerging topics included telepractice in dysphagia management (9%, *n* = 2) and electrostimulation in dysphagia treatment (9%, *n* = 2).

Decade‐based comparison indicated variation in topic focus over time. Studies in the 1990s focused primarily on autonomy and informed consent. Studies in the 2000s and 2010s included additional topics such as cultural considerations and shared decision‐making. Studies in the 2020s addressed a wider range of topics, including telepractice and systemic equity.

### 3.5 Ethical Principles

The frequency of ethical principles reported across decades is summarised in Table 2. Across all decades, autonomy was the most consistently represented principle, appearing in every period reviewed. In the 1990s (*n* = 6 studies), autonomy (*n* = 4) and informed consent (*n* = 3) predominated, with more limited attention to beneficence (*n* = 2), justice (*n* = 2) and non‐maleficence (*n* = 1). During the 2000s, autonomy remained prominent(*n* = 5), alongside beneficence (*n* = 2), justice (*n* = 2) and informed consent (*n* = 2), while non‐maleficence continued to be infrequently addressed (*n* = 1). The 2010s were characterised by a narrow ethical focus, with only three studies identified, addressing autonomy (*n* = 2) and informed consent (*n* = 1). In contrast, the 2020s demonstrated a broader ethical engagement, with autonomy again most frequent (*n* = 5), accompanied by increased attention to beneficence (*n* = 3), justice (*n* = 3), non‐maleficence (*n* = 2) and informed consent (*n* = 2). Detailed decade‐specific distributions are presented in Table 2.

**TABLE 2:** Descriptive results indicating the approach followed, principles addressed, ethical component highlighted and the role of the SLT addressed.

Author(s)	Year	Journal	Country/region	Approach/methodology	Topic focus	Principle	Ethical component	Role of SLT
Serradura‐Russell	1992	*Dysphagia* Q1 IF 3.2	Australia	Social (case study approach)	Autonomy in dysphagia care	Autonomy, informed consent	Judgement	Management
Sharp and Genesen	1996	*American Journal of Speech‐Language Pathology* Q1 IF 3.1	United States	Social (case study approach)	Ethical dilemmas in dysphagia	Beneficence, non‐maleficence	Judgement	Management
Kirschner and Sorties	1996	*Top Stroke Rehabil* Q1 IF 2.8	United States	Social (case study approach)	Ethical decision‐making post‐stroke	Autonomy, justice	Sensitivity	Assessment, management
Brady and Pittenger	1997	*Swallowing and Dysphagia Disorders* Q4 IF 0.975	United States	Social (case study approach)	Case‐based ethical analysis	Autonomy, beneficence	Judgement	Assessment, management
Lefton‐Greif and Arvedson	1997	*Seminars in Speech and Language* Q2 IF 1	United States	Philosophical (principle‐based approach)	Paediatric dysphagia	Informed consent, justice	Judgement	Advocacy
Landes	1999	*American Journal of Speech‐Language Pathology* Q1 IF 3.1	United States	Philosophical (principle‐based approach)	Advocacy in dysphagia management	Autonomy, informed consent	Motivation	Advocacy
O'Toole	2000	*American Journal of Speech‐Language Pathology* Q1 IF 3.1	United States	Social (case study approach)	Ethical decision‐making in dysphagia	Informed consent, shared decision‐making (autonomy)	Sensitivity	Advocacy counselling
Hemsley and Balandin	2003	*Advances in Speech‐Language Pathology* Q1 IF 2.1	Australia	Philosophical (principle‐based approach)	Communication in dysphagia management	Autonomy	Sensitivity	Advocacy counselling
Sharp and Bryant	2003	*Seminars in Speech and Language* Q2 IF 1	United States	Philosophical (principle‐based approach)	Ethics when patients refuse care	Autonomy and beneficence	Judgement	Management counselling
Sharp	2006	*Top Stroke Rehabilitation* Q1 IF 2.8	United States	Philosophical (principle‐based approach)	Dysphagia management after stroke	Informed consent	Judgement	Advocacy counselling education
Arvedson & Lefton‐Greif	2007	*Seminars in Speech and Language* Q2 IF 1	United States	Philosophical (principle‐based approach)	Ethical decision‐making in paediatric dysphagia	Evidence‐based practice (beneficence, non‐maleficence, justice, autonomy)	Motivation	Advocacy
Sharp and Wagner	2007	*Topics in geriatric rehabilitation* Q3 IF 0.7	United States	Philosophical (principle‐based approach)	Ethics, informed consent and decisions about nonoral feeding for patients with dysphagia	Autonomy, beneficence and shared decision‐making	Sensitivity	Management counselling
Huffman and Owre	2008	*Language, Speech and Hearing Services in Schools* Q1 IF 3.2	United States	Social (case study approach)	Ethics in school‐based dysphagia services	Justice and autonomy	Judgement	Assessment management education
Kenny	2015	*Bioethics* Q1 IF 1.98	Australia	Social (case study approach)	Food culture and ethics in dysphagia	Shared decision‐making (autonomy)	Sensitivity	Management
Horner et al.	2016	*American Journal of Speech‐Language Pathology* Q1 IF 3.1	United States	Philosophical (principle‐based approach)	Consent and refusal in dysphagia care	Informed consent	Judgement	Advocacy management counselling
Kelly et al.	2018	*International Journal of Speech‐Language Pathology* Q1 IF 1.9	Australia	Philosophical (principle‐based approach)	Ethical and legal Aspects in palliative dysphagia	Shared decision‐making (autonomy)	Motivation	Management
Leslie and Lisiecka	2020	*Seminars in Speech and Language* Q2 IF 1	United Kingdom/Ireland	Philosophical (principle‐based approach)	Ethics in complex dysphagia	Autonomy and justice	Judgement	Management
Matos et al.	2022	*BMC Neurology* Q2 IF 2.2	Brazil	Social (Case study approach)	Electrostimulation in dysphagia	Informed consent	Action	Management
Ward et al.	2022	*Dysphagia* Q1 IF 3.2	Global	Philosophical (principle‐based approach)	Telepractice in dysphagia management	Justice, autonomy, beneficence	Sensitivity	Identification assessment management consultation
O'Keeffe et al.	2023	*BMC Medical Ethics* Q1 IF 3.1	Global	Philosophical (principle‐based approach)	Informed consent and modified texture diets	Shared decision‐making (autonomy) and duty of care (beneficence, non‐maleficence and justice.)	Sensitivity	Management advocacy consultation
Weaver and Geppert	2023	*Journal of Pain and Symptom Management* Q1 IF 4.7	United States	Social (case study approach)	Ethical dilemmas in dysphagia	Shared decision‐making (autonomy)	Sensitivity	Management counselling consultation
Cimoli et al.	2024	*Frontiers in Rehabilitation Sciences* Q2 IF 1.9	United States/Australia	Philosophical (principle‐based approach)	Dysphagia management and unintended consequences of NPO	Informed consent, autonomy, non‐maleficence	Judgement	Advocacy management counselling consultation education

### 3.6 Ethical Components

The ethical components identified across the included studies mapped closely onto Rest's Four‐Component Model, reflecting differing emphases on the psychological processes underlying ethical behaviour in dysphagia management (Table 3).

**TABLE 3:** Integration of ethical approaches, principles and Rest's components identified across studies.

Ethical approach	Dominant ethical principles	Linked rest component(s)	Illustrative decade	Example study focus
Philosophical /principle‐based	Autonomy, beneficence, justice	Moral judgment, moral motivation	Strong in 1990s–2000s; resurged 2020s	Conceptual analyses of patient choice and clinician responsibility
Case‐based / applied	Autonomy, non‐maleficence, informed consent	Moral sensitivity, moral action	Increased from 2000s onward	Practical dilemmas in feeding decisions, aspiration risk, telepractice
Hybrid / contextual (minor subset)	Beneficence + justice	Moral sensitivity + judgment	Emerging in 2020s	Equity and access in telehealth and palliative dysphagia care

Across the full dataset, moral judgment was the most frequently represented component, appearing in 45% (*n* = 10) of the 22 studies. This component was consistently evident across decades and reflects a strong focus in the literature on evaluating ethical dilemmas and determining appropriate courses of action. Moral sensitivity was the second most commonly identified component, present in 36% (*n* = 8) of studies, indicating attention to recognising ethically salient features of clinical situations. In contract moral motivation was less frequently addressed, appearing in 14% (*n* = 3) of studies, while moral action was identified in only one study (5%), representing the least frequently examined component across the corpus.

Overall, the distribution of ethical components demonstrates a predominant focus on the cognitive and perceptual aspects of ethical reasoning (moral sensitivity and moral judgment), with comparatively limited attention to the prioritisation and enactment of ethical decisions (moral motivation and moral action). The relationships between ethical approaches, principles and Rest's components are synthesised in Table 3 to support interpretability.

### 3.7 Role of the SLT

The roles of SLTs in dysphagia management were diverse and multifaceted across the included studies (Table 2). Management was the most frequently addressed role, appearing in 77% (*n* = 17) of the 22 studies. Advocacy was identified in 41% (*n* = 9) of studies, while counselling featured in 36% (*n* = 8)

Less frequently reported roles included assessment, education, research, consultation and administration, reflecting a broad but uneven distribution of professional responsibilities represented in the literature. Most studies described SLTs occupying multiple roles within a single ethical context, rather than restricting ethical considerations to a single aspect of practice.

Across the dataset, role distributions showed consistent alignment with ethical emphases identified elsewhere in the review. Management‐ and counselling‐focused roles most commonly co‐occurred with discussions of autonomy and moral judgment, whereas advocacy roles were more frequently associated with justice and components related to moral motivation and moral action. These associations are presented descriptively and summarised in Table 2.

When examined temporally, foundational clinical roles such as assessment and management appeared consistently across decades, while roles related to advocacy, education, administration and telepractice emerged more prominently in later publications. Given the small number of studies per decade, these temporal patterns are reported descriptively, with decade‐specific role distributions presented in Table 2.

Interpretation of how evolving SLT roles intersect with ethical principles, psychological components of ethical behaviour and implications for practice and education is addressed in the discussion.

## 4 Discussion

This scoping review examined ethical challenges in dysphagia management through the combined lenses of principled ethics and Rest's Four‐Component Model. The findings reveal a persistent structural imbalance in the literature. Ethical discourse is dominated by autonomy and informed consent, alongside the cognitive components of moral sensitivity and moral judgment, while moral motivation and moral action remain comparatively underdeveloped. This imbalance helps explain the recurring tension between principle‐based recommendations and their implementation in everyday clinical practice.

### 4.1 Ethical Principles, Ethical Reasoning and Practice Tensions

The centrality of autonomy and informed consent across the literature reflects the profession's longstanding commitment to person‐centred care. In dysphagia management, decisions about texture modifications, non‐oral feeding or risk‐aligned eating frequently intersect with patients’ cultural identities, social participation and quality of life. As such, autonomy functions not merely as an abstract principle but as a lived ethical challenge that requires negotiation between clinical safety and personal meaning.

Recent studies illustrate how these ethical tensions manifest in contemporary practice. Refusal of texture‐modified diets, continued oral intake despite aspiration risk, and disagreements about non‐oral feeding compel clinicians to balance autonomy, beneficence, and non‐maleficence in contexts shaped by institutional policies and medico‐legal concerns (O'Keeffe et al. 2023; Weaver and Geppert 2023). While philosophical analyses tend to advocate for respect of informed refusal, case‐based studies more often highlight organisational constraints that complicate enactment of such principles. Together, these findings suggest that ethical challenges in dysphagia care are less about uncertainty regarding what is ethically appropriate and more about how ethical commitments are operationalised within complex clinical systems.

### 4.2 Insights from Rest's Four‐Component Model

Applying Rest's Four‐Component Model (1994) provides process‐oriented understanding of this implementation gap. The predominance of moral sensitivity and moral judgment indicates that SLTs are well equipped to recognise ethical dilemmas and reason through competing values. However, the relative absence of moral motivation and moral action suggests limited attention to how ethical priorities are sustained and enacted under real‐world pressures.

Moral motivation requires clinicians to prioritise ethical values—such as autonomy, dignity and equity—over competing influences such as time constraints, hierarchical decision‐making or fear of litigation. Moral action extends this process by translating ethical intent into practice, often requiring advocacy or resistance to institutional norms. The underrepresentation of these components likely reflects the realities of multidisciplinary and medicalised care environments, where ethical reasoning may be acknowledged but not readily enacted. Without explicit support for moral motivation and action, clinicians may experience moral distress or default to defensive practices, even when ethical awareness is strong. This finding underscores the need for ethics education and service structures that extend beyond ethical reasoning to include advocacy skills, reflective supervision and institutional support mechanisms.

### 4.3 Ethical Evolution and Contemporary Challenges

Rather than reflecting linear ethical progress, the temporal patterns identified in this review suggest shifting emphases shaped by broader healthcare changes. Early literature focused on foundational ethical principles, while later work increasingly engages with contextual complexities such as shared decision‐making, cultural diversity and service accessibility. More recent studies reflect emerging ethical challenges linked to technological innovation and systemic inequities, particularly through telepractice.

Telepractice exemplifies the dual ethical potential identified in the literature. On one hand, it advances justice by improving access to dysphagia services for underserved populations. On the other, it introduces new ethical risks related to equity, privacy, informed consent and digital exclusion (Ward et al. 2022; Smart et al. 2023). Ethical implementation therefore requires explicit consideration of technological risks, patient capacity to engage digitally and safeguards to ensure confidentiality and informed choice. Viewed through Rest's model, telepractice places particular demands on moral motivation and action, as clinicians must proactively address inequities rather than merely recognise them.

### 4.4 Cultural Ethics, Shared Decision‐Making and Global Contexts

Cultural awareness and shared decision‐making emerged as central mechanisms through which ethical principles are enacted in practice. Respecting cultural food practices, language preferences and family decision‐making structures operationalises autonomy, beneficence and justice simultaneously. These practices align closely with moral sensitivity and moral judgment but also require moral motivation and action when cultural values conflict with institutional norms or risk‐averse practices.

It is important to acknowledge that both Beauchamp and Childress’ principlism and Rest's Four‐Component Model are grounded in Western ethical traditions. While these frameworks offer valuable structure for ethical analysis, their application across diverse global contexts warrants reflexivity. In LMIC settings, ethical decision‐making is often shaped by resource constraints, collective decision‐making norms, and systemic inequities that individualistic ethical models may not fully capture. Recognising these limitations reinforces the importance of contextualised ethics scholarship and the inclusion of non‐Western perspectives in future research.

## 5 Synthesis and Implications

Collectively, the findings indicate that ethical practice in dysphagia management is characterised by strong ethical awareness and reasoning, but more limited attention to the behavioural and motivational dimensions of ethical action. This imbalance has practical consequences: ethical principles are widely endorsed, yet their enactment is frequently constrained by organisational, cultural and systemic factors. Strengthening ethical practice therefore requires moving beyond principle recognition toward supporting clinicians in translating ethical commitments into action.

Future research should examine how moral motivation and moral action develop in clinical contexts, particularly through interprofessional ethics education, reflective supervision and organisational policy. By addressing both cognitive and behavioural components of ethical practice, the profession can better support SLTs in delivering care that is not only ethically informed but ethically enacted.

## 6 Implications for Practice

The findings of this review underscore that ethical competence in dysphagia management extends beyond ethical awareness and principled reasoning to include the capacity to act ethically within complex clinical and organisational contexts. Cultural awareness, shared decision‐making and person‐centred communication are not ancillary skills but practical mechanisms through which autonomy, beneficence and justice are enacted in everyday care.

The observed imbalance between cognitive ethical components (moral sensitivity and judgment) and behavioural components (moral motivation and action) has clear implications for practice. While SLTs are well positioned to recognise ethical dilemmas and articulate principled responses, they may lack the institutional authority, interprofessional support or practical tools required to translate ethical intent into action—particularly in contexts involving risk‐aligned eating, capacity disputes or telepractice delivery.

Addressing this gap requires service‐level supports that enable ethical action, including structured shared decision‐making processes, early ethics consultation in contested cases and explicit organisational policies that support autonomy‐informed risk‐taking where decision‐making capacity is established. In emerging service models such as telepractice, ethical implementation must attend not only to access and efficiency but also to equity, informed consent, privacy and patient agency. Strengthening these practical supports is essential to ensuring that ethical principles are not only recognised but consistently realised in dysphagia care.

## 7 Limitations and Future Directions

This review underscores the need for more inclusive research that reflects the context specific challenges and systemic constraints experienced by SLTs in LMICs. The existing literature remains predominantly shaped by high‐income countries, limiting the transferability of ethical frameworks and practice recommendations to under‐resourced settings. Future research should prioritise culturally diverse and resource‐sensitive perspectives to ensure broader applicability of ethical guidance in dysphagia management.

Several methodological considerations should also be acknowledged. As a scoping review, no formal risk‐of‐bias or quality appraisal was conducted and the included studies varied widely in design, ranging from conceptual analyses to single‐case reports. In addition, the relatively small number of studies per decade and the possibility of publication bias limit the strength of inferences regarding temporal shifts. Consequently, observed shifts in ethical emphasis should be interpreted cautiously, as they may reflect publication patterns rather than definitive changes in professional practice. Future systematic or mixed‐methods reviews incorporating formal quality assessment may strengthen the evidence base and enable more robust conclusions.

The exclusion of literature published prior to 1980, particularly non‐digitised or hard‐copy sources, represents a further limitation. While this review intentionally focuses on contemporary developments in dysphagia ethics, this criterion may have excluded earlier foundational contributions that informed subsequent ethical discourse.

Although Rest's Four‐Component Model provided a useful theoretical lens, the limited representation of moral motivation and moral action within the included studies reflects a gap in the current literature rather than a limitation of the framework itself. Further empirical research is needed to explore how these components are enacted in clinical practice and supported within organisational and interprofessional contexts.

Finally, it should be noted that both reviewers were also authors of the study. To mitigate potential interpretive bias, independent coding and structured consensus discussions were employed throughout the review process. Nonetheless, some degree of residual bias cannot be entirely excluded and should be considered when interpreting the findings.

## 8 Conclusion

This review demonstrates that ethical discourse in dysphagia management has been shaped predominantly by autonomy and informed consent, with increasing but uneven attention to beneficence, justice and non‐maleficence. Applying Rest's Four‐Component Model highlights a persistent imbalance within literature: while moral sensitivity and moral judgment are well developed, moral motivation and action remain comparatively underrepresented. This distinction between ethical awareness (principles, moral sensitivity and moral judgment) and ethical operationalisation (moral motivation and moral action) represents a critical leverage point for improving ethical practice, shifting the focus from knowing what is ethically appropriate to being able to enact it within real‐world clinical and organisational constraints.

Emerging themes, including cultural awareness, shared decision‐making and telepractice, illustrate how ethics in dysphagia care is becoming increasingly complex and context dependent. These themes interact directly with Rest's model by revealing that recognising ethical dilemmas and reasoning about them is insufficient unless clinicians are also supported to prioritise ethical values and translate them into action, particularly in situations involving risk‐aligned eating, capacity disputes and technologically mediated care.

Addressing this gap requires coordinated action across professional domains. Clinicians should embed structured ethical reflection into routine decision‐making, actively integrate patient values into care planning and seek support when ethical tensions arise. Education and training programmes should prioritise applied ethics approaches—such as case‐based learning, simulation and interprofessional dialogue—to strengthen moral motivation and ethical action. At a systems level, professional bodies and policymakers should support ethical practice through clear guidance, accessible ethics consultation mechanisms, and telepractice policies that promote equity, informed consent and patient safety.

The findings also underscore the importance of global and contextual sensitivity in ethical practice. The predominance of evidence from high‐income countries limits the applicability of existing ethical frameworks to LMICs, where resource constraints, cultural practices and service delivery models may differ substantially. Advancing ethical practice in dysphagia management therefore requires context‐sensitive research and policy development that support not only ethical awareness, but also the motivation and structural capacity to act ethically across diverse healthcare environments.

## Conflicts of Interest

The authors declare no conflicts of interest.

## Data Availability

All data generated or analysed during this study are included in this published article.
